# Effectiveness of thermal radiations and homogeneous–heterogeneous reactions in Maxwell flow field across a rotating cylinder

**DOI:** 10.1016/j.heliyon.2023.e20381

**Published:** 2023-09-21

**Authors:** Shuguang Li, Jawad Ahmed, Faisal Nazir, Showkat Ahmad Lone, Mohamed Abdelghany Elkotb

**Affiliations:** a School of Computer Science and Technology, Shandong Technology and Business University, Yantai 264005, China; b Department of Basic Sciences, University of Engineering and Technology, Taxila 47050, Pakistan; c Department of Basic Sciences, College of Science and Theoretical Studies, Saudi Electronic University, Riyadh 11673, Saudi Arabia; d Mechanical Engineering Department, College of Engineering, King Khalid University, Abha 61421, Saudi Arabia

**Keywords:** Maxwell fluid, Rotating cylinder, Solar radiations, Homogeneous–heterogeneous reactions, Numerical solution

## Abstract

This paper investigates the impact of cubic autocatalysis on energy transport in Maxwell fluid flow induced into a rotating cylinder inspired by a solar radiative surface. The homogeneous-reaction is assumed to be furnished by the kinetics of isothermal cubic autocatalytic and the heterogeneous reaction by kinetics of first order. To prevent induced axial secondary flow, the cylinder's rotation is maintained at a constant rate. The characteristics of thermal radiation are also investigated to regulate the pace of heat transmission. A magnetic beam is projected in the upward radial direction to control the fluid momentum. A suitable flow ansatz is used to convert the entire physical problem of thermal energy transmission and fluid flow from partial differential equations (PDEs) to nonlinear ordinary differential equations (ODEs). Results obtained numerically with the bvp4c approach are presented graphically and explained physically. It is observed that by flourishing the Reynolds parameter, the penetration depth decreases. Further, when the thermal relaxation period increases, the temperature field degrades. Moreover, when the homogeneous–heterogeneous reaction's strength is increased, a reduction in fluid concentration is shown.

## 1 Introduction

Scientists have been attracted to the topic of non-Newtonian liquid flow because it has so many engineering applications, including the processing of adhesive tapes, coating applications, and glass blowing, all of which frequently call for the flow of non-Newtonian liquids above rigid surfaces. Since each non-Newtonian fluid has unique properties, no single mathematical relationship can adequately describe the flow behaviour of completely non-linear fluids. In Refs. [[1], [2], [3], [4]], flows of non-Newtonian liquids were described for a variety of geometries and physical suppositions. Dhinakaran et al. [5] used the Phan-Thien-Tanner model to study the steady movement of viscoelastic fluid among parallel plates while taking electro-osmotic forces into account. Utilizing MHD theory, Prasad et al. [6] looked at the viscoelastic flow of a fluid with heat transfer and varying viscosity over a stretching sheet. According to their investigation, the surface temperature gradient was decreased by higher magnetic parameter values. The lattice Boltzmann approach was used by Malaspinas et al. [7] to investigate viscoelastic fluid that were both linear and non-linear. Siddiqa et al. [8] numerically analyzed the non-Newtonians fluid-free convection flow over a vertical surface. In their investigation, displacement thickness and dimensionless velocities were examined for various viscoelastic parameter values. Some non-Newtonian fluid flows over various configurations can be seen in Refs. [[9], [10], [11], [12], [13], [14], [15]].

Viscoelastic fluids display the viscous and elastic effects of non-linear fluids, and their stress-strain relationship is time-dependent. To mathematically explain the stress relaxation process in fluids, the Maxwell model has been suggested. The majority of materials used in industries exhibit viscoelastic fluid behaviour under stress, as in the production of plastics, paints, polymers, and rubber sheets. Research on the Maxwell fluid flow between parallel plates by Wenchang et al. [16] was reported. The problem was resolved using the Laplace and Fourier transforms. Abbasbandy et al. [17] investigated the Falkner-Skan flow of MHD Maxwell fluid. Both analytical and numerical solutions were reported in this work. An immiscible fractional Maxwell fluid's multi-layer flows were studied by Rauf et al. [18]. They used the Laplace transform in conjunction with the zero-order Hankel and Weber transform to arrive at the problem's analytical solution. It was demonstrated that the velocity falls with increasing fractional parameter values. According to Khan et al. [19], the Maxwell fluid flow around a stretching horizontal spinning cylinder when subjected to a transverse magnetic field is investigated. Khan et al. [20] analyzed thermal energy transfer processes in Maxwell fluid flow with the use of Cattaneo-Christov theory.

Many applications, ranging from axles and shafts to spinning projectiles, depend on the flow over rotating cylinders. The incompressible flow around a revolving circular cylinder has recently been the subject of extensive research utilizing computational, theoretical, and experimental techniques. Engineering structures and aerodynamic problems are just two examples of this field's many uses. Rotational cylinders and other control techniques, including Lorentz forces, blowing, suction, and surface roughness, can be used to regulate boundary layer flows. Crane [21] was the first to study the 2D flow produced by stretching a cylinder. Ishak and Nazar [22] were able to determine the numerical and accurate solutions for the axisymmetric fluid flow close to the stretching cylinder. The flow over a permeable stretched cylinder was examined by Mastroberardino and Paullet [23]. Fang and Yao [24] examined the axisymmetric viscous flow over a revolving and extending cylinder. Sprague and Weidman [25] investigated how a rotating cylinder affected viscous fluid flow. Khan et al. [26] recently modelled and simulated the Maxwell fluid resulting from cylinder torsion. Thakur et al. [27] projected the heat transfer in the flow caused by the revolving cylinder contained by the non-Newtonian fluid of power-law type. The properties of Newtonian thermal effects in the flow of nanofluids through a porous cylinder were investigated by Hayat et al. [28]. In this work, the modelled problem has been resolved using a semi-analytical method (HAM). In Refs. [[29], [30], [31]], the reader can learn more about the flow of nanofluid when a cylinder is stretched.

It is emphasized that research on the analysis of homogeneous–heterogeneous reactions in the flow of Maxwell fluid induced by a rotating cylinder was not concentrated in light of the aforementioned rotational flows. Understanding the rheology of a three-dimensional (3D) Maxwell fluid boundary layer flow with homogeneous–heterogeneous reactions over a rotating cylinder is the primary goal of the current work. The energy transport characteristics are investigated when non-linear thermal radiations occur for fluid motion of Maxwell model. The physical problem is modelled in the form of PDEs, which are then changed into a collection of non-linear ODEs by applying the proper flow similarities. The numerical calculations of the generated ODEs are performed using the MATLAB bvp4c methodology. The obtained data are graphically displayed together with a physical explanation.

## 2 Problem formulation

**Fig. 1:**
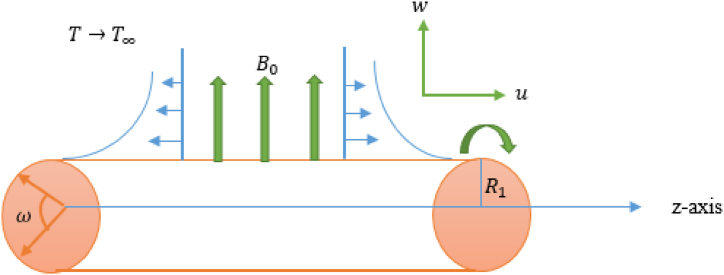
Flow configuration.

Suppose a horizontally rotating and stretchable cylinder with a radius of $R_{1}$ that induces a Maxwell fluid (electrically conducting) swirling under the impact of magnetic flux. Assuming that the cylinder rotates around its axis at a constant speed and that the axial distance has a linear relationship with the cylinder's stretching velocity. It is assumed that the velocity field for the flow is given by the equation V=(u,v,w), where u,v,w are the components along the *z*-, ω-, and r-axes, correspondingly. The magnetic field that is applied uniformly and in the direction of the r-axis is denoted by the formula $B=\left(0,0,B_{0}\right)\text{.}$ By assuming that $T\left(z,R_{1}\right)=T_{w}$ for the temperature at the cylinder's surface, thermal analysis is examined. Fig. 1 provides the geometry of the physical model.

There is a consideration for homogeneous–heterogeneous reactions in Maxwell fluid motion. When cubic autocatalysis occurs, the homogeneous reaction is

(1) $$A+2B\to 3B,\;r=k_{c}ab^{2}\text{,}$$

For the isothermal first-order reaction to the form, we have

(2) $$A\to B,\;r=k_{s}a.$$

In Eqs. (1), (2), ($k_{c},k_{s}$) are the rate constants, (A,B) the chemical species, and (a,b) the concentrations of those species. Moreover, we presume that this chemical reaction is isothermal. The governing equations of the Maxwell 3D flow in the presence of the magnetic field chemical reactions, and nonlinear thermal radiations are:

(3) ∇·V=0,

(4) $$\rho \left(V.\nabla \right)V=-\nabla p+\nabla \cdot \;S+J_{1}\times B\text{,}$$

(5) $$\rho c_{p}\left(V.\nabla \right)T=k\nabla^{2}T\text{,}$$

(6) $$\left(V.\nabla \right)a=D_{A}\nabla^{2}a-k_{c}ab^{2}\text{,}$$

(7) $$\left(V.\nabla \right)b=D_{B}\nabla^{2}b+k_{c}ab^{2}\text{.}$$

For the Maxwell fluid, **S** is the extra stress tensor defined as

(8) $$\left(1+\lambda_{1}\frac{D}{Dt}\right)S=\mu A_{1}\text{,}$$

where $\lambda_{1}$ is the time of relaxation, and $\frac{D}{Dt}$ shows the upper convective derivative, *μ* the dynamic viscosity of liquid. In Eq. (8), the Newtonian fluid condition can be recovered when $\lambda_{1}$ = 0. Further

$A_{1}$ indicates the first Rivlin-Ericksen tensor written as

(9) $$A_{1}=\nabla V+{\left(\nabla V\right)}^{t}\text{,}$$

and

(10) $$\frac{DS}{Dt}=\frac{\partial S}{\partial t}+\left(V.\nabla \right)S-LS-SL^{t}\text{.}$$

In the equations above, $c_{p}$ for heat capacity at constant pressure, t for transpose, $J_{1}$ for current density, $D_{A}$ and $D_{B}$ are the diffusion coefficients. Moreover, T and $T_{\infty }$ stand for fluid temperature and the free stream temperature. According to the steady, incompressible and axisymmetric supposition, the governing Eqs. (3), (4), (5), (6), (7) in view of Eqs. (8), (9), (10) for the current energy and flow transfer problem under non-linear radiations are given below (Refs. [9,19]).

(11) $$\frac{\partial u}{\partial r}+\frac{\partial w}{\partial z}+\frac{u}{r}=0\text{,}$$

(12) $$u\frac{\partial u}{\partial z}+w\frac{\partial u}{\partial r}+\lambda_{1}\left(u^{2}\frac{\partial^{2}u}{\partial z^{2}}+2uw\frac{\partial^{2}u}{\partial r\partial z}+w^{2}\frac{\partial^{2}u}{\partial r^{2}}\right)=\nu \left(\frac{\partial^{2}u}{\partial r^{2}}+\frac{1}{r}\frac{\partial u}{\partial r}\right)-\frac{\sigma {B_{0}}^{2}}{\rho }\left(u+\lambda_{1}w\frac{\partial u}{\partial r}\right)\text{,}$$

(13) $$u\frac{\partial v}{\partial z}+w\frac{\partial v}{\partial r}+\frac{wv}{r}+\lambda_{1}\left(u^{2}\frac{\partial^{2}v}{\partial z^{2}}+2uw\frac{\partial^{2}v}{\partial r\partial z}+w^{2}\frac{\partial^{2}v}{\partial r^{2}}+\frac{2wv}{r}\frac{\partial w}{\partial r}+\frac{2uv}{r}\frac{\partial w}{\partial z}-\frac{2w^{2}v}{r^{2}}\right)=\nu \left(\frac{\partial^{2}v}{\partial r^{2}}-\frac{v}{r^{2}}+\frac{1}{r}\frac{\partial v}{\partial r}\right)-\frac{\sigma {B_{0}}^{2}}{\rho }\left(v+\lambda_{1}w\frac{\partial v}{\partial r}-\lambda_{1}\frac{wv}{r}\right)\text{,}$$

(14) $$u\frac{\partial T}{\partial z}+w\frac{\partial T}{\partial r}=\alpha_{1}\left(\frac{\partial^{2}T}{\partial r^{2}}+\frac{1}{r}\frac{\partial T}{\partial r}\right)-\frac{1}{\rho c_{p}}\frac{1}{r}\frac{\partial }{\partial r}\;\left(rq_{r}\right)\text{,}$$

(15) $$u\frac{\partial a}{\partial z}+w\frac{\partial a}{\partial r}=D_{A}\left(\frac{\partial^{2}a}{\partial r^{2}}+\frac{1}{r}\frac{\partial a}{\partial r}\right)-k_{c}ab^{2}\text{,}$$

(16) $$u\frac{\partial b}{\partial z}+w\frac{\partial b}{\partial r}=D_{B}\left(\frac{\partial^{2}b}{\partial r^{2}}+\frac{1}{r}\frac{\partial b}{\partial r}\right)+k_{c}ab^{2}\text{,}$$

with conditions

(17) $$u=2\alpha z,v=E,w=0,T=T_{w},D_{A}\frac{\partial a}{\partial r}=k_{s}a,D_{B}\frac{\partial b}{\partial r}={-k}_{s}a\;\text{at}\;r=R_{1}\text{,}$$

(18) $$u=0,\;v=0,\;T\to T_{\infty },\;a\to a_{0},\;b\to 0\;\text{as}\;\text{r}\to \infty \text{.}$$

Here, v stands for kinematic viscosity, σ for fluid electric conductivity, $\alpha_{1}$ for thermal diffusivity, $B_{0}$ for magnetic field strength, and α(>0) for cylinder stretching strength having dimension $T^{-1}$. Moreover, $q_{r}=-\frac{16\sigma^{*}}{3k^{*}}T^{3}\frac{\partial T}{\partial r}$ is the non-linear radiative heat flux, $k^{*}$ is the absorption coefficient, and $\sigma^{*}$ is the Stefan-Boltzmann constant, respectively. The following transformation group is introduced (Refs. [9,19]):

(19) $$\eta =\frac{r^{2}}{R_{1}^{2}},\;u=2\alpha zf^{'}\left(\eta \right),\;v=Eg\left(\eta \right),\;w=-\alpha R_{1}\frac{f\left(\eta \right)}{\eta^{\frac{1}{2}}}\text{,}$$

(20) $$\theta \left(\eta \right)=\frac{T-T_{\infty }}{T_{w}-T_{\infty }},\;\varphi \;\left(\eta \right)=\frac{a}{a_{0}},\;\psi \left(\eta \right)=\frac{b}{a_{0}}.$$

When the preceding ansatz in Eqs. (19), (20) is used, Eq. (11) automatically is satisfied, while Eqs. (12), (13), (14), (15), (16), (17), (18) yield.

(21) $$\eta f^{\prime \prime \prime }+f^{\prime \prime }+Reff^{\prime \prime }-{Ref}^{\prime 2}-\beta_{1}Re\left(\frac{f^{2}f^{\prime \prime }}{\eta }+2f^{2}f^{\prime \prime \prime }-4ff^{\prime }f^{\prime \prime }\right)-MRe\left(\frac{f^{\prime }}{2}-\beta_{1}ff^{\prime \prime }\right)=0\text{,}$$

(22) $$2\eta^{2}g^{\prime \prime }+2\eta g^{\prime }-\frac{g}{2}+2Re\eta fg^{\prime }+Refg-\beta_{1}Re\left(2f^{2}g^{\prime }+4\eta f^{2}g^{\prime \prime }+4ff^{\prime }g-\frac{4f^{2}g}{\eta }\right)-MRe\left(\eta g-{2\beta }_{1}\eta fg^{\prime }-\beta_{1}fg\right)=0\text{,}$$

(23) $${\left(1+R_{d}{\left(1+\left(\theta_{w}-1\right)\theta \right)}^{3}\right)(\eta \theta }^{\prime \prime }+\theta^{\prime })+RePrf\theta^{\prime }=0\text{,}$$

(24) $$\frac{1}{S_{c}}\left(\eta \varphi^{\prime \prime }+\varphi^{\prime }\right)+Re\left(f\varphi^{\prime }-k_{1}\varphi \psi^{2}\right)=0\text{,}$$

(25) $$\frac{\delta }{S_{c}}\left(\eta \psi^{\prime \prime }+\psi^{\prime }\right)+Re\left(f\psi^{\prime }+k_{1}\varphi \psi^{2}\right)=0\text{,}$$

with conditions

$$f^{'}\left(1\right)=1,\;g\left(1\right)=1,\;f\left(1\right)=0,\;\theta \left(1\right)=1,\;\varphi^{'}\left(1\right)=k_{2}\varphi \left(1\right),\;\delta \psi^{'}\left(1\right)=-k_{2}\varphi \left(1\right)\text{,}$$

(26) $$f^{'}\left(\infty \right)=0,\;g\left(\infty \right)=0,\;\theta \left(\infty \right)=0,\;\varphi \left(\infty \right)=1,\;\psi \left(\infty \right)=0.$$

In the above equations, $\beta_{1}=\lambda_{1}\alpha$ is the Maxwell number, $M=\frac{\sigma {B_{0}}^{2}}{\alpha \rho }$ is the magnetic number, $Re=\frac{\alpha R_{1}^{2}}{2v}$ is the Reynolds number, $\mathrm{Pr}=\frac{v}{\alpha_{1}}$ is Prandtl number, $\theta_{w}=\frac{T_{w}}{T_{\infty }}$ is the temperature ratio parameter, $R_{d}=\frac{16\sigma^{*}T_{\infty }^{3}}{3kk^{*}}$ is the radiation parameter, $k_{1}=\frac{k_{c}a_{0}^{2}}{2a}$ homogeneous reaction, $k_{2}=\frac{k_{s}R_{1}}{2D_{A}}$ the heterogeneous reaction parameter, $S_{c}=\frac{v}{D_{A}}$ is the Schmidt parameter, and $\delta =\frac{D_{B}}{D_{A}}$ is the diffusion coefficients ratio.

Now, assume that the diffusion coefficients ($D_{A},D_{B}$) are equal in size. This hypothesis leads to an examination of chemical reactions when $D_{A}$ and $D_{B}$ are equal, i.e., δ=1. The relationship that results from this assumption is as follows:

(27) ψ(η)=1−φ(η).

Using Eq. (27), Eqs. (24), (25) transform into

(28) $$\frac{1}{S_{c}}{(\varphi^{'}+\eta \varphi }^{''})+Re\left[f\varphi^{'}-\;k_{1}\varphi {\left(1-\varphi \right)}^{2}\right]=0\text{,}$$

with conditions

(29) $$\varphi^{\prime }\left(1\right)=k_{2}\varphi \left(1\right),\varphi \left(\infty \right)=1\text{.}$$

The solution of the flow equation converges too slowly, especially for lesser values of Re, as reported by Fang and Yao [24]. Thus, the variable η is converted as $\eta =e^{x}$ in order to follow Fang and Yao for achieving fast convergence. Hence, Eqs. (21), (22), (23), (26), (28), (29) become

(30) $$f_{xxx}-2f_{xx}+f_{x}-Re\left(f_{x}^{2}-ff_{xx}+ff_{x}\right)-\beta_{1}Ree^{-x}\left(2f^{2}f_{xxx}-5f^{2}f_{xx}+3f^{2}f_{x}-4ff_{x}f_{xx}+4ff_{x}^{2}\right)-MRe\left(e^{x}\frac{f_{x}}{2}-\beta_{1}ff_{xx}+\beta_{1}ff_{x}\right)=0,$$

(31) $$2g_{xx}-\frac{g}{2}+Re\left(2fg_{x}+fg\right)-\beta_{1}Ree^{-x}\left(2f^{2}g_{x}+4f^{2}g_{xx}+4f^{2}g_{x}+4ff_{x}g-4f^{2}g\right)-MRe\left(e^{x}g-2\beta_{1}fg_{x}-\beta_{1}gf\right)=0\text{,}$$

(32) $$\left(1+R_{d}{\left(1+\left(\theta_{w}-1\right)\theta \right)}^{3}\right)\theta_{xx}+RePrf\theta_{x}=0\text{,}$$

(33) $${\frac{1}{S_{c}}\varphi }_{xx}+Re\left(f\varphi_{x}-e^{x}k_{1}\varphi {\left(1-\varphi \right)}^{2}\right)=0\text{,}$$

with the transformed BCs

$$f\left(0\right)=0,f_{x}\left(1\right)=1,g\left(0\right)=1,\theta \left(0\right)=1,\varphi_{x}\left(0\right)=k_{2}\varphi \left(0\right)\text{,}$$

(34) $$f_{x}\left(\infty \right)=0,g\left(\infty \right)=0,\theta \left(\infty \right)=0,\varphi \left(\infty \right)=1\text{.}$$

## 3 Numerical solution

In this part, numerical solutions to well-known ODEs that reflect the energy and flow transport equations (30), (31), (32), (33) and BCs in equation (34) are proposed. The numerical findings are acquired using MATLAB's built-in bvp4c approach. The three-stage Lobatto III formula, along with the finite difference technique, is used for improving initial guesses and is known as built-in bvp4c. However, with the implementation of the collocation formula generates a $C^{1}$-continuous polynomial solution with the fourth order as the default accuracy in the defined interval. The residual of the solution is a key component of the mesh and error control. The bvp4c strategy requires transforming the governing ODEs into a system of first-order ODEs by using the modified variables as $f=y_{1}$, $f_{x}=y_{2}$, $f_{xx}=y_{3}$, $f_{xxx}=yy_{1}$, $g=y_{4}$, $g_{x}=y_{5}$, $g_{xx}=yy_{2}$, $\theta =y_{6}$, $\theta_{x}=y_{7},\;\theta_{xx}=yy_{3}$, $\varphi =y_{8}$, $\varphi_{x}=y_{9},\;\mathrm{and}\;\varphi_{xx}=yy_{4}$, for Eqs. (30), (31), (32), (33), (34). The first-order ODEs that result are listed below in Eqs. (35), (36), (37), (38), (39):

(35) $$yy_{1}=\left(2y_{3}-y_{2}+Re\left(y_{2}^{2}-y_{1}y_{3}+y_{1}y_{2}\right)+\beta_{1}Ree^{-x}\left(3y_{1}^{2}y_{2}-5y_{1}^{2}y_{3}-4y_{1}y_{2}y_{3}+4y_{1}y_{2}^{2}\right)+MRe\left(e^{x}\frac{y_{2}}{2}-\beta_{1}y_{1}y_{3}+\beta_{1}y_{1}y_{2}\right)\right)/a_{1}\text{,}$$

(36) $$yy_{2}=\left(\frac{y_{4}}{2}-Re\left(2y_{1}y_{5}+y_{1}y_{4}\right)+\beta_{1}Ree^{-x}\left(6y_{1}^{2}y_{5}+4y_{1}y_{2}y_{4}-4y_{1}^{2}y_{4}\right)+MRe\left(e^{x}y_{4}-2\beta_{1}y_{1}y_{5}-\beta_{1}y_{1}y_{4}\right)\right)/a_{2}\text{,}$$

(37) $$yy_{3}=-RePry_{1}y_{7}/b_{1}\text{,}$$

(38) $$yy_{4}=-ReS_{c}y_{1}y_{9}+Re\;S_{c}e^{x}k_{1}y_{8}{\left(1-y_{8}\right)}^{2}\text{,}$$

where

$$a_{1}=1-2\beta_{1}Ree^{-x}y_{1}^{2},a_{2}=2-4\beta_{1}Ree^{-x}y_{1}^{2},b_{1}=\left(1+R_{d}{\left(1+\left(\theta_{w}-1\right)y_{6}\right)}^{3}\right)\text{,}$$

with conditions

$$y_{1}\left(0\right)=0,\;y_{2}\left(0\right)=1,\;y_{4}\left(0\right)=1,\;y_{6}\left(0\right)=1,\;y_{9}\left(0\right)=k_{2}y_{8}\left(0\right)\text{,}$$

(39) $$y_{2}\left(\infty \right)=0,y_{4}\left(\infty \right)=0,y_{6}\left(\infty \right)=0,y_{8}\left(\infty \right)=1\text{.}$$

## 4 Discussion of results

In this section, we compute the numerical outcomes in the form of graphical depictions for flow and heat transmission while considering the variations in physical parameters, such as the Reynolds parameter Re, magnetic parameter M, Maxwell number $\beta_{1}$, Prandtl parameter Pr, radiation number $R_{d}$, Schmidt number Sc, temperature ratio parameter $\theta_{w}$, homogeneous reaction parameter $k_{1}$, and heterogeneous reaction number $k_{2}$. The parameters are varied in the ranges as 3.0≤Re≤6.0, 2.0≤M≤5.0, $0.01\leq \beta_{1}\leq 0.15$, 4.0≤Pr≤7.0, $0.3\leq R_{d}\leq 0.9$, $0.5\leq \theta_{w}\leq 1.4$, $1.0\leq S_{c}\leq 1.6$, $0.1\leq k_{1}\leq 0.7$, $0.7\leq k_{2}\leq 1.3$. The values of the parameters are fixed throughout the entirety of the computational study as Re=3.0, M= 2.0, $\beta_{1}=0.01$, Pr=5.0, $R_{d}=0.7$, $S_{c}=1.5$, $\theta_{w}=1.5,{\text{ k}}_{\text{1}}{\text{=k}}_{\text{2}}\text{=0.1.}$.

**Fig. 2:**
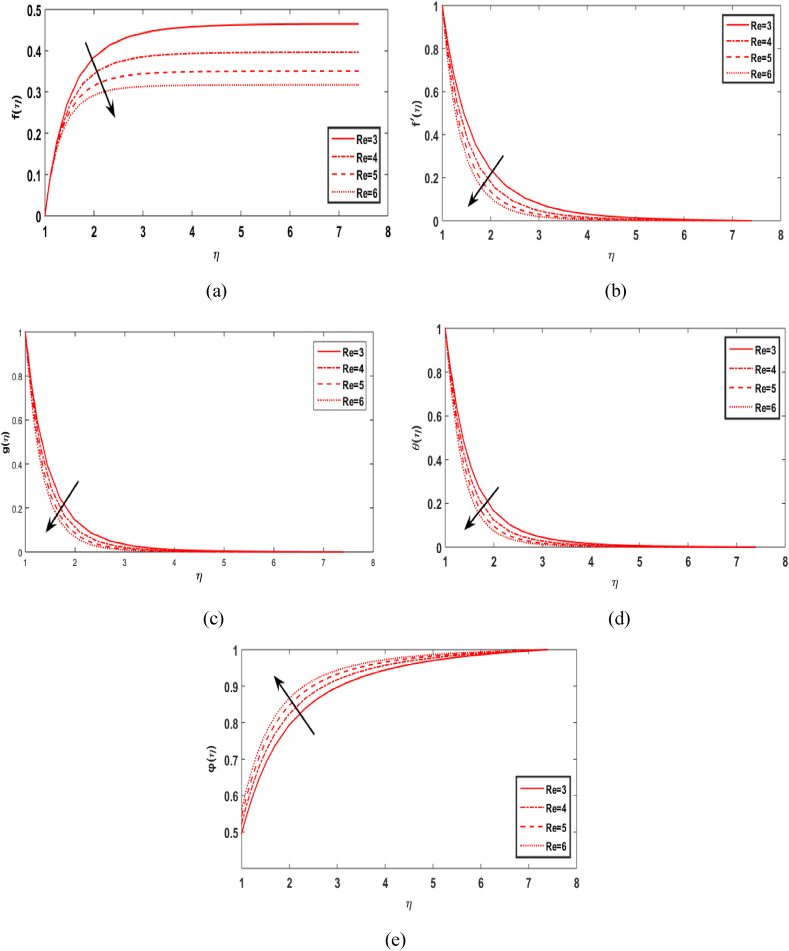
Effect of Re over f(η), $f^{\prime }\left(\eta \right)$, g(η), θ(η) and φ(η).

Fig. 2(a)-2(e) show the impact of Reynolds Re versus radial velocity f(η), axial velocity $f^{\prime }\left(\xi \right)$, tangential velocity distribution g(ξ), temperature profile θ(ξ), and concentration distribution φ(η). It is observed that the flow field declines and fluid movement only takes place near the cylinder's surface. As the value of Re upsurge then the axial, radial and tangential velocities of the liquid decrease. Physically, greater values of Re cause an upsurge in the inertial force to the flow of the field. The fluid flow agent is opposed by the inertial force, which causes the flow field's velocity to decrease in all directions. It is perceived from graph 2(d) of temperature field θ(η) that as the value of Re rises, the temperature profile decreases. Physically, the forced convection process in the flow is known to be reduced by a greater value of Re, which results in a decrease in the temperature field. The concentration distribution φ(η) increases as parameter variation Re increases, as depicted in Fig. 2(e).

**Fig. 3:**
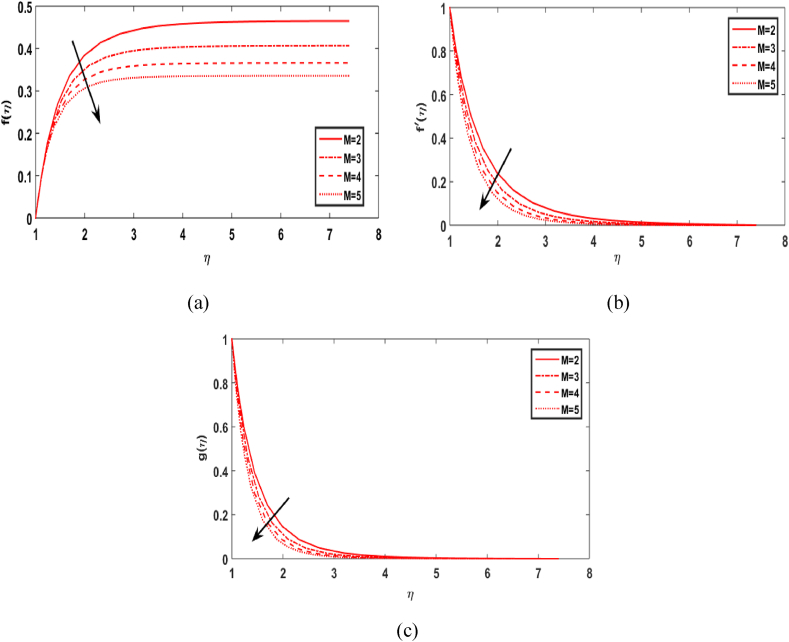
Effect of M over f(η), $f^{\prime }\left(\eta \right)$, and g(η).

Fig. 3(a)-3(c) represent the influence of the magnetic number M on the radial, axial and tangential velocities. As M increases, the radial velocity f(η), axial velocity field $f^{\prime }\left(\eta \right)$ and tangential velocity distribution g(η) all decrease. The primary cause is that a magnetic field's presence produces a resistive force called the Lorentz force that slows fluid velocity.

Fig. 4(a)-4(b) discuss the influence of Maxwell number $\beta_{1}$ on the radial velocity f(η) and axial velocity field $f^{\prime }\left(\eta \right)$. The higher value of Maxwell's number drops both the radial and axial velocity field components. From a physical point of view, the stress relaxation effects in a viscoelastic fluid are accelerated by higher values of $\beta_{1}$, which leads to the observed fall in the velocity field of Maxwell fluid over a rotating cylindrical surface.

**. 4:**
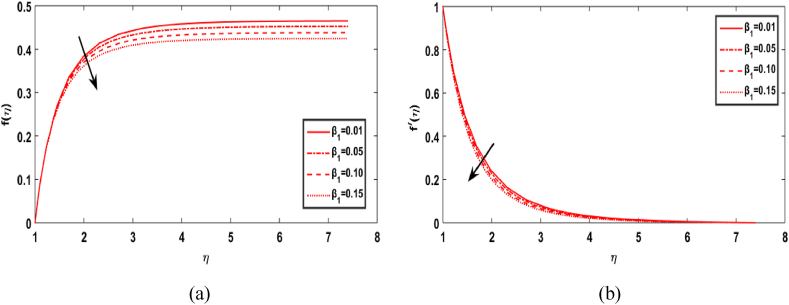
Effect of $\beta_{1}$ over f(η) and $f^{\prime }\left(\eta \right)$.

**Fig. 5:**
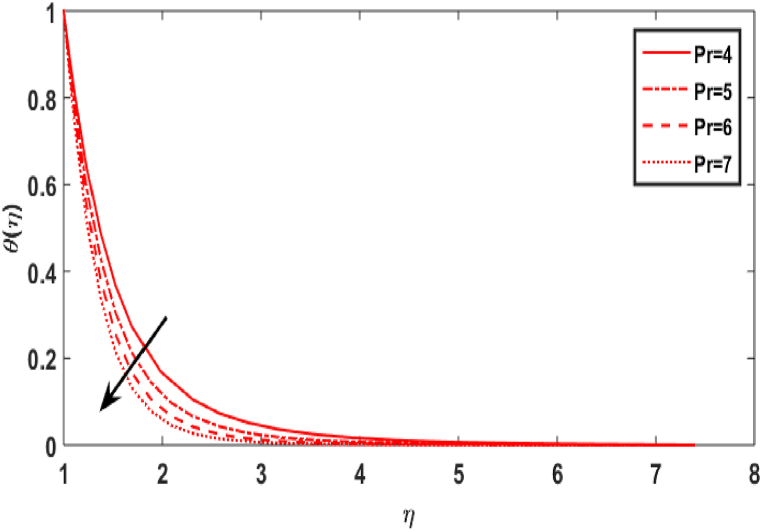
Effect of Pr over θ(η).

**Fig. 6:**
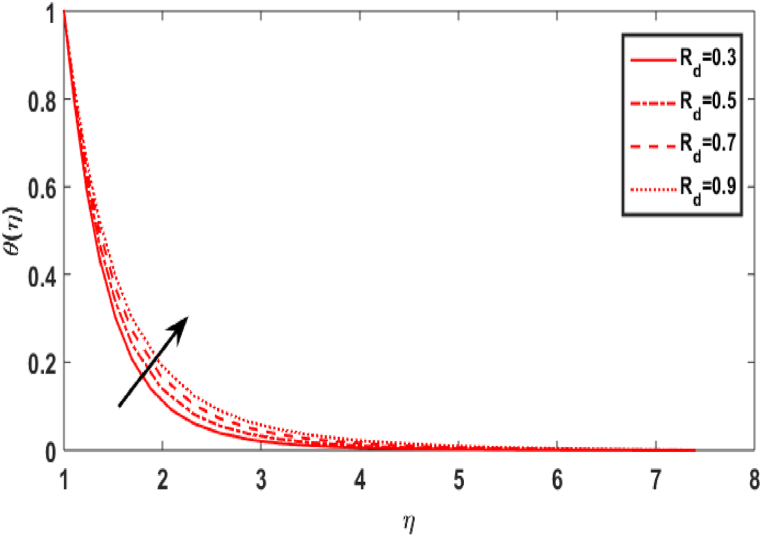
Effect of $R_{d}$ over θ(η).

**Fig. 7:**
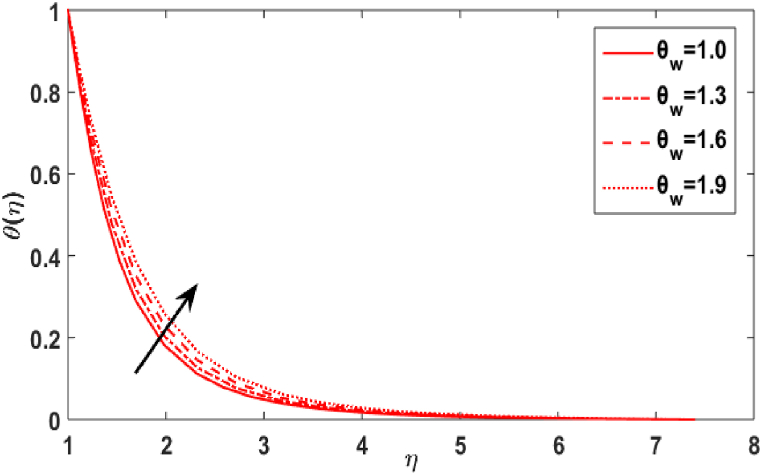
Effect of $\theta_{w}$ over θ(η).

Fig. 5 depicts the result of the Prandtl number Pr on the temperature distribution. According to Fig. 5, with increasing values, the Prandtl number Pr has a decreasing relationship with the temperature distribution of transmission in the fluid flow. Physically, when, $\mathrm{Pr}=\frac{v}{\alpha_{1}}$ increases, $\alpha_{1}$ decreases, i.e., the fluid's thermal diffusivity lowers. Consequently, as Pr improves, the heat transmit through the fluid diminishes. Fig. 6 symbolises the impact of the radiation parameter $R_{d}$ against temperature field. It is obvious from the sketch that when the value of radiation parameter upsurges, the temperature distribution increases. The existence of a radiative parameter suggests that a fluid would absorb more heat when its value is higher. Fig. 7 illustrates the value of temperature ratio parameter $\theta_{w}$ against the temperature distribution. Physically, the graph of the temperature field uplifts when the value of the temperature ratio number rises.

**Fig. 8:**
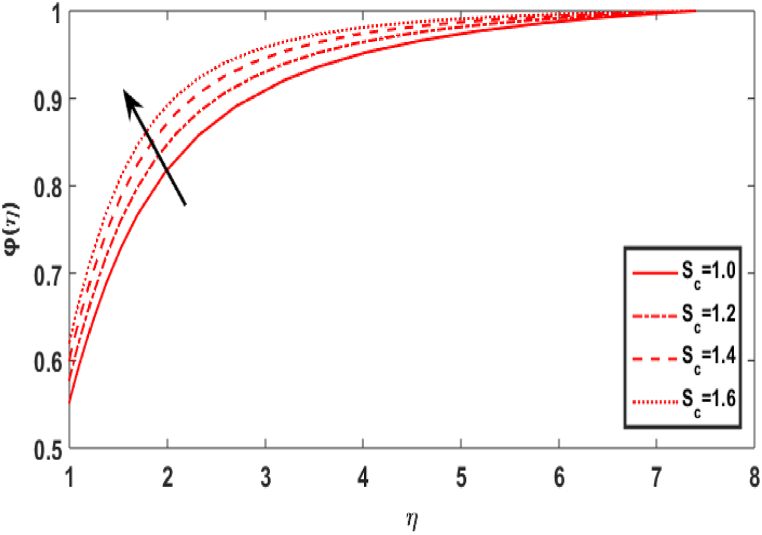
Effect of $S_{c}$ over φ(η).

**Fig. 9:**
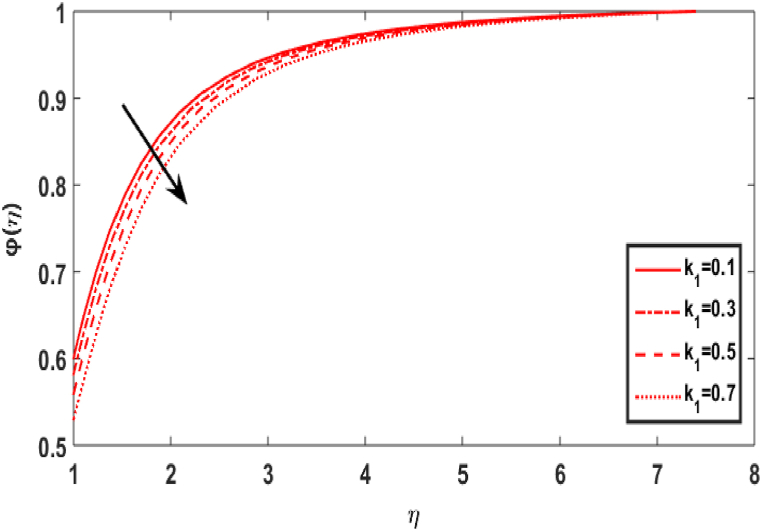
Effect of $k_{1}$ over φ(η).

**Fig. 10:**
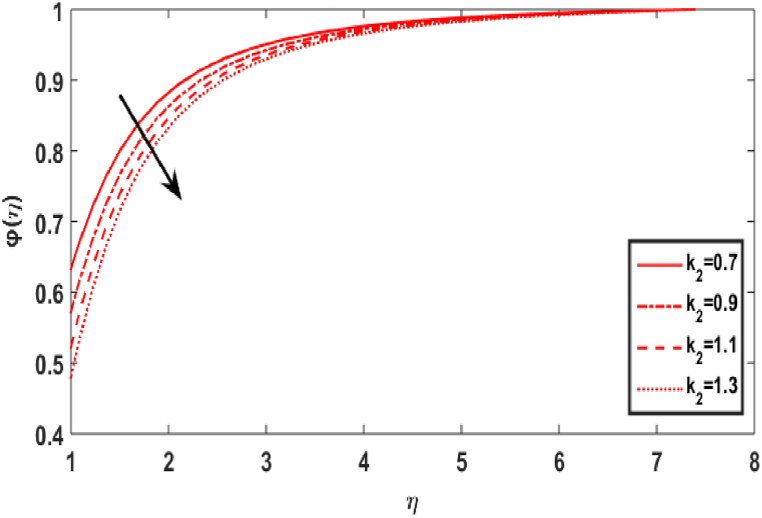
Effect of $k_{2}$ over φ(η).

Fig. 8 shows the effect of Schmidt number $S_{c}$ on the concentration field φ(η). The Schmidt parameter $S_{c}$ is the ratio of momentum over mass diffusivities. The concentration profile appears to improve with increasing Schmidt numbers. The upsurge $S_{c}$ relates to decrease in mass diffusivity which leads an increase in fluid concentration. Fig. 9 divulges the upshot of homogeneous reaction number $k_{1}$ on the concentration distribution φ(η). This is caused by the response rates overwhelming the diffusion coefficients. The cumulating values of $k_{1}$ decrease the graph of concentration. Fig. 10 displays the variation in concentration distribution φ(η) for the various values of the heterogeneous parameter $k_{2}$. It is evident that as the value of heterogeneous reactions increases, the concentration decreases. This develops because the rise in $k_{1}$ or $k_{2}$ indicates that reactants are being consumed in the flow field, which corresponds to a drop in the concentration field.

## 5 Conclusions

The aim of the present work was to study the Maxwell fluid flow across a rotating cylinder under the action of magnetic flux. Further, the thermal transport features were explored in relation to non-linear thermal radiation. The novelty of this work was the existence of homogeneous–heterogeneous chemical reactions on the concentration profile. The transformed physical model was tackled numerically with the help of the MATLAB solver ‘bvp4c’. The key parameters were varied to anticipate their consequences on the dimensionless profiles of Maxwell fluid velocity, temperature, and concentration. The following were the main findings of our current work.

• The three velocity components were all decreased in the presence of a magnetic field in each of their respective directions, which raised the temperature of the fluid.
• It was discovered that the homogeneous and heterogeneous numbers both had a diminishing effect on the concentration boundary layer thickness.
• By raising the Schmidt parameter $S_{c}$, considerable growth was seen in the concentration profile.
• The temperature of the fluid was lowered as a result of growing the Prandtl and Reynolds numbers.
• When the Maxwell parameter was increased, the concentration and thermal fields increased, but the field of flow decreased.
• In order to reduce the concentration distribution, it was shown to be highly beneficial to consider the effects of homogeneous and heterogeneous chemical reaction parameters.

## Author contribution statement

Shuguang Li: Analyzed and interpreted the data; Wrote the paper. Jawad Ahmed: Conceived and designed the experiments; Wrote the paper. Faisal Nazir: Performed the experiments; Wrote the paper. Showkat Ahmad Lone: Contributed reagents, materials, analysis tools or data. Mohamed Abdelghany Elkotb: Conceived and designed the experiments

## Data availability statement

No data was used for the research described in the article.

## Declaration of competing interest

The authors declare that they have no known competing financial interests or personal relationships that could have appeared to influence the work reported in this paper.

## List of symbols

u,v,w: Velocity components $\left[ms^{-1}\right]$
r,ω,z: Polar coordinates [m]
B: Magnetic field
$B_{o}$: Strength of magnetic field $\left[NmA^{-1}\right]$
$c_{p}$: Specific heat capacity $\left[JK^{-1}kg^{-1}\right]$
ρ: Density of fluid $\left[kgm^{-3}\right]$
$\beta_{1}$: Maxwell parameter
M: Magnetic field number
Pr: Prandtl parameter
Re: Reynolds parameter
$R_{d}$: Radiation parameter
T: Fluid temperature [K]
$T_{\infty }$: Ambient Temperature [K]
$T_{w}$: Wall temperature [K]
$D_{A}$, $D_{B}$: Diffusion coefficients
$S_{c}$: Schmidt parameter
δ: Diffusion coefficients ratio parameter
$k_{1}$: Homogeneous reaction parameter
$k_{2}$: Heterogeneous reaction parameter
$f^{\prime }$: Axial velocity
f: Radial velocity
g: Azimuthal velocity
ν: Kinematic viscosity $\left[m^{2}s^{-1}\right]$
σ: Electric conductivity of the fluid $\left[Sm^{-1}\right]$
η: Dimensionless variable
θ: Temperature profile
φ: Concentration profile
V: Velocity field
μ: Dynamic viscosity $\left[kgm^{-1}s^{-1}\right]$
$\alpha_{1}$: Thermal diffusivity $\left[m^{2}s^{-1}\right]$
$\lambda_{1}$: Fluid relaxation parameter $\left[T^{-1}\right]$
$\theta_{w}$: Temperature ratio parameter
α: Stretching rate $\left[T^{-1}\right]$

## References

[1] Naccache M.F., Mendes P.R.S. (1996). Heat transfer to non-Newtonian fluids in laminar flow through rectangular ducts. Int. J. Heat Fluid Flow.

[2] Shin S. (1996). The effect of the shear rate-dependent thermal conductivity of non-Newtonian fluids on heats transfer in a pipe flow. Int. Commun. Heat Mass Tran..

[3] Nouri J.M., Whitelaw J.H. (1997). Flow of Newtonian and non-Newtonian fluids in an eccentric annulus with rotation of the inner cylinder. Int. J. Heat Fluid Flow.

[4] Attia H.A. (2005). Numerical study of flow and heat transfer of non-Newtonian fluid on a rotating porous disk. Appl. Math. Comput..

[5] Dhinakaran S., Afonso A.M., Alves M.A., Pinho F.T. (2010). Steady viscoelastic fluid flow between parallel plates under electro-osmotic forces: Phan-Thien-Tanner model. J. Colloid Interface Sci..

[6] Prasad K.V., Pal D., Umesh V., Rao N.S.P. (2010). The effect of variable viscosity on MHD viscoelastic fluid flow and heat transfer over a stretching sheet. Comm. Nonl. Sci. Nume. Simul..

[7] Malaspinas O., Fietier N., Deville M. (2010). Lattice Boltzmann method for the simulation of viscoelastic fluid flows. J. Non-Newt. Fluid Mech..

[8] Siddiqa S., Begum N., Hossain M.D.A., Gorla R.S.R. (2017). Natural convection flow of a two-phase dusty non-Newtonian fluid along a vertical surface. Int. J. Heat Mass Tran..

[9] Ahmed A., Khan M., Ahmed J. (2020). Thermal analysis in swirl motion of Maxwell nanofluid over a rotating circular cylinder. Appl. Math. Mech. -Engl. Ed..

[10] Ahmed J., Khan M., Rasheed Z., Ahmed A., Ahmed L., Bhandari A. (2022). Entropy generation analysis for axisymmetric flow of carreau nanofluid over a horizontally stretching cylinder. Proce. Instit. Mech. Eng., Part E: J. Proc. Mech. Eng.

[11] Khan M., Hafeez A., Ahmed J. (2022). Von Karman swirling flow of an Oldroyd‐B nanofluid with the influence of activation energy. Math. Meth. Appl.Sci..

[12] Shatanawi W., Abbas N., Shatnawi Taqi A.M., Hasan F. (2023). Heat and mass transfer of generalized Fourier and Fick's law for second-grade fluid flow at slendering vertical Riga sheet. Heliyon.

[13] Rehman S., Alfaleh A., Afef K., Hashim, Shah S.I.A. (2023). Onset about isothermal flow of Carreau liquid over converging channel with Cattaneo-Christov heat and mass fluxes. Heliyon.

[14] Qayyum M., Riaz M.B., Afzal S. (2023). Analysis of blood flow of unsteady Carreau-Yasuda nanofluid with viscous dissipation and chemical reaction under variable magnetic field. Heliyon.

[15] Zafar S.S., Khan U., Ali F., Eldin S.M., Saeed A.M., Zaib A., Galal A.M. (2023). Irreversibility analysis of radiative flow of Prandtl nanofluid over a stretched surface in Darcy-Forchheimer medium with activation energy and chemical reaction. Heliyon.

[16] Wenchang T., Wenxiao P., Mingyu X. (2003). A note on unsteady flows of a viscoelastic fluid with the fractional Maxwell model between two parallel plates. Int. J. Non-Linear Mec..

[17] Abbasbandy S., Naz R., Hayat T., Alsaedi A. (2014). Numerical and analytical solutions for Falkner-Skan flow of MHD Maxwell fluid. Appl. Math. Comput..

[18] Rauf A., Mahsud Y., Siddique I. (2019). Multi-layer flow of immiscible fraction al Maxwell fluids in a cylindrical domain. Chin. J. Phy..

[19] Khan M., Ahmed A., Ahmad J. (2020). Boundary layer flow of Maxwell fluid due to torsional motion of cylinder: modeling and simulation. Appl. Math. Mech.-Engl. Ed...

[20] Khan M., Ahmad A., Irfan M., Ahmed J. (2021). Analysis of Cattaneo–Christov theory for unsteady flow of Maxwell fluid over stretching cylinder. J. Therm. Anal. Calor.

[21] Crane L.J. (1975). Boundary layer flow due to a stretching cylinder. Z. Ange. Math Phy..

[22] Ishak A., Nazar R. (2009). Laminar boundary later flow along stretching cylinder. Eur. J. Sci. Res..

[23] Mastroberardino A., Paullet J.E. (2010). Existence and a priori bounds for steady stagnation flow toward a stretching cylinder. J. Math. Anal. Appl..

[24] Fang T., Yao S. (2011). Viscous swirling flow over a stretching cylinder. Chin. Phys. Lett..

[25] Sprague M.A., Weidman P.D. (2011). Three-dimensional flow induced by the torsional motion of the cylinder. Flu. Dyn. Res..

[26] Khan M., Ahmed A., Ahmed J. (2020). Boundary layer flow of Maxwell fluid due to torsional motion of cylinder. Appl. Math Mech. Engl. Ed..

[27] Thakur P., Tiwari N., Chhabra R.P. (2019). Momentum and heat transfer from an asymmetrically confined rotating cylinder in a power-law fluid. Int. J. Ther. Sci..

[28] Hayat T., Khan M.I., Waqas M., Alsaedi A. (2017). Newtonian heating effect in nanofluid flow by a permeable cylinder. Res. Phys..

[29] Imtiaz M., Hayat T., Alsaedi A. (2016). Mixed convection flow of Casson nanofluid over a stretching cylinder with convective boundary conditions. Adv. Powd. Tech..

[30] Khan M., Irfan M., Khan W.A. (2019). Heat transfer enhancement for Maxwell nanofluid flow subject to convective heat transport. Pram.

[31] Dhanai R., Rana P., Kumar L. (2016). MHD mixed convection nanofluid flow and heat transfer over an inclined cylinder due to velocity and thermal slip effects. Powd. Tech..

